# Boosting inhibition control process by knitting at school

**DOI:** 10.3389/fpsyg.2023.1062001

**Published:** 2023-06-23

**Authors:** Frederic Sonnier, Emeline Lussiana, Sabine Gueraud

**Affiliations:** UFR Psychologie, Université Paris 8, Saint-Denis, France

**Keywords:** executive function, inhibition, learning, knitting, emotion

## Abstract

Across two experiments, the presented research explored the impact of a knitting bout on elementary school pupils’ inhibition abilities. They proposed an accurate measure of the pupils’ inhibition abilities through the use of a stop-signal paradigm. In order to take into account, the differentiation between cool and hot inhibitions abilities, the emotional content of the stimuli was manipulated across experiments. Neutral materials were used in Experiment 1 when emotionally charged materials were in Experiment 2. The findings of both experiments highlighted a beneficial impact of the knitting bout on children’s inhibition abilities. While the results of Experiment 1 showed an optimization of inhibition abilities for the knitting session group in comparison to the control group, Experiment 2 revealed a disappearance of the effect of the emotional content on these abilities as well. Proposals as to why EF could be sensitive to knitting practice are discussed.

## Introduction

Executive function (EF) is conceptualized as cross-cutting processes that are exerted on task-specific controlled processes and are thought to intervene during complex or new behaviors, that is when automatic processes are not sufficient to deal with the situation in hand. Within their componential EF framework, Miyake et al. (2000) pointed to three essential EF components: inhibition, updating of working memory and mental flexibility. They highlighted, through confirmatory factorial analyses, the distinct nature of these executive processes albeit they cannot be considered entirely independent. This distinction in the implementation and functioning of these processes has called for a fractionated rather than a general study of cognitive control (Gallant, 2016). Furthermore, while EF was initially investigated with emotionally neutral material, certain models claimed for a distinction between two types of EF depending on the nature of information it operates on. The so-called hot EF is assumed to operate on emotionally charged information whereas the so-called cool one on neutral information (Zelazo and Müller, 2002; Zelazo and Carlson, 2012). This differentiation is supported by developmental findings that demonstrated independent and distinct developmental trajectory for each EF type. According to Zelazo and Carlson (2012), both develop in a linear manner but the development of the hot EF is delayed compared to the cool EF (Carlson, 2005). Other authors describe a quadratic development of hot EF with a specific pattern in adolescents (Casey, 2015; Aïte et al., 2016).

Previous research has demonstrated that EF is heavily involved in many mental activities, such as problem-solving (Clément, 2006), categorization (Blaye and Jacques, 2009) and emotional regulation (Carlson and Wang, 2007; Sperduti et al., 2017). EF has been found to be omnipresent in school tasks (Meltzer, 2018) and appears to be impaired in individuals suffering from pervasive developmental disorders (Diamond et al., 1997; Valeri and Speranza, 2009). It has also been shown EF is paramount in the management of conflict and interference (Friedman and Miyake, 2004), that is in the management of attentional resources shared between the execution and control of the task in hand (Engle et al., 1999; Miyake et al., 2000; Barrouillet and Camos, 2001; Cowan, 2016). Based on the core role of EF in everyday behaviors from childhood to adulthood, psychological research pursues two main objectives, understanding how EF develops and how it can be improved. The present research focused on the latter purpose with 9–11 years old children. More specifically, its purpose was to investigate the conditions under which executive processes could be optimized within ecological school settings. It does not focus however on academic activities but rather in activities that do not directly sustain academic learning. In other words, it seeks to explore which activities practice within a class or school may foster children’s executive capacities so that they could be placed in a more favorable cognitive state for academic learning.

Among the new non-academic activities introduced into school, knitting caught our attention. Knitting consists of making with needles a fabric made of a textile material arranged in mesh. Knitting is considered as a manual activity of artisanal and artistic creation. The simplicity of the material, two needles and wool, its portability and its flexibility facilitate the practice. It is an activity that is easy to learn in the early stages and can become complex at leisure. Knitting involves movements that require both hands, are rhythmic, repetitive and can become automatic with expertise (Corkhill et al., 2014). In a psychological perspective, knitting possess two properties of interest. Like any manual activity, knitting is both process-oriented and outcome-oriented (Blanche, 2007). And, it is a task that involves both motor and cognitive skills since knitters have to master a precise sequence of gestures while maintain their attention over the duration in order to scrupulously follow the various stages of the realization. For the learner, this activity requires special concentration and sustained attention.

The few studies that have been conducted to explore the impact of knitting practice on the cognitive sphere of individuals suggest that this practice could have an impact on EF (Hartzell et al., 2021). Studies in children reported an impact of knitting practice on the involvement, the concentration and the attentional capacities of students (Beloshistaya and Zhukova, 2008). In addition, within the field of the knitting activity, some authors claimed for a likeness between attentional focused meditation (AF meditation)—which is a type of meditation that consists of intense, purposeful concentration on an object of attention such as one’s own breathing—and knitting practice in non-expert as knitting is a manual activity that requires sustained concentration and fine motor skills in a calm group setting in the sense that it entails mastering a sequence of precise gestures and maintaining attention over a period of time (Corkhill et al., 2014). The repetitive nature of knitting is thought to “create a space for contemplation” and bring about a “state of enhanced calm” (Turney, 2009, p. 152; see also, Riley et al., 2013; Alhers and Thomas, 2019). For example, a study of 3,545 adults who regularly knit showed that focusing attention on bilateral gestures and the object of creation induces a state of calmness and serenity in people (Riley et al., 2013). Similarly, the recent research of Rusiñol-Rodriguez et al. (2020) highlighted the hypnotic, contemplative and relaxing nature of this activity. Since one major finding of the impact of AF mediation and mindfulness is their beneficial effects on EF in both adults (Moore and Malinowski, 2009; Gallant, 2016; Luu and Hall, 2017; Cásedas et al., 2019) and children and adolescents (Thurman and Torsney, 2014; Kiani et al., 2017), on may expect that knitting practice could be favorable for EF as well. The purpose of the present research was then to investigate this claim, that is, whether a non-academic activity like knitting could have an impact on schoolchildren aged 9–11 EF. However, we did not examine EF as a whole but focused on the inhibition component of EF only for two reasons. Firstly, inhibition abilities are considered fundamental in an individual’s cognitive functioning insofar as their integrity is essential to maintaining a satisfactory level of adaptation in a constantly changing environment (Simpson and Riggs, 2007; Verbruggen and Logan, 2008) and are assumed to support other executive abilities functioning (Pennington, 1997; Carlson and Moses, 2001; Blair et al., 2005). Secondly, the work of Houdé and colleagues highlighted the essential role of inhibition control in cognitive development and learning (Borst et al., 2015) as it has been shown to be determinant in the development of complex cognitive skills such as reasoning, arithmetic (Houdé and Borst, 2015), decision making (Cassotti et al., 2014) or creativity (Cassotti et al., 2016).

The concept of inhibition encompasses a number of dimensions (Logan, 1994; Nigg, 2000; Friedman and Miyake, 2004; Aron, 2007; Garon et al., 2008) ranging on a continuum from behavioral inhibition (i.e., blocking of preponderant responses) to conceptual inhibition (i.e., selection and retention of information according to its relevance). Because it is difficult to differentiate experimentally between inhibition acting on mental representations and cognitive processes and inhibition modulating motor responses (Chevalier, 2010), the current research deals with behavioral inhibition. According to Logan and Cowan (1984), the inhibition of dominant actions (i.e., behavioral inhibition) can be seen as an interaction between a high-level executive system that manages objectives and goal changes and a subordinate system that is responsible for its application. These authors suggested that simple actions depend on the activation of routines requiring little attentional control. However, when there is conflict between several actions and when the semi-automatic process for resolving this conflict is not sufficient, the executive system intervenes to orient the choice of action schemas. In experimental studies of the management of motor responses and their inhibition, two paradigms are classically used, namely the go/no-go (Donders, 1969) and the stop-signal (Lappin and Eriksen, 1966; Logan and Cowan, 1984) paradigms. Both are based on a binary choice reaction task, the most common of which is a categorization task (i.e., letters or arrow direction). They differ from one another in terms of the motor association with the categories of stimuli. In a go/no-go task, one category is associated with a motor response (i.e., pressing a key, Go), while the other is not (i.e., No-Go). However, Verbruggen and Logan (2008) showed that in this type of task, inhibition can be explained by the competition between two automatic memory processes (the go response and the stop response) without the control process intervention. The literature has thus evidenced a preference in recent years for the stop-signal paradigm (Urben, 2011). In contrast to the go/no-go paradigm, in a stop-signal paradigm, each category of stimuli is associated with an action. In a quarter of the trials, a stop signal is given shortly after the stimulus indicating to participants that they should stop their response. In this case, no memory association is possible, so the inhibition processes are controlled processes. The stop-signal paradigm therefore allows to study as closely as possible the processes involved in inhibitory control (Verbruggen and Logan, 2008; Urben et al., 2014).

The current research then used a Strop-Signal task to evaluate whether knitting practice at school promote performance on a task involving the participants’ inhibition abilities.

To address this question, two experiments were designed across which the emotional content of the stimuli was manipulated in order to take into account the distinction between cool inhibitory capacities (affectively neutral) and hot inhibitory capacities (affectively charged). In Experiment 1, a cool stop-signal paradigm embedded within a pre-test—activity—post-test protocol was used. In Experiment 2 both a neutral and an emotionally charged stop-signal tasks were performed by participants, within a counterbalanced activity—stop-signal task design, to explore whether the positive impact of knitting observed in Experiment 1 on cool inhibition abilities expanded into hot ones as well. In both Experiment 1 and 2, an experimental group, which took part in a knitting session, was compared with a control group, which had a recess session. Recess was chosen as the control condition because it traditionally represents a time of relaxation at school, a break and a source of learning about social relationships and child culture (Delalande, 2009) that is far removed from the sphere of academic cognitive activities. Nevertheless, all the pupils included in the research were taught how to knit by voluntary workers from outside the school in fortnightly sessions over a period of 3 months before the experiments in order to guarantee the absence of any interference associated with the benefits that such an activity might have in the long term.

## Experiment 1

The objective of Experiment 1 was to examine the acute effects of knitting at school on 9 to 11-year-old pupils’ cool inhibitory abilities. It investigated the impact of a knitting bout on behavioral inhibition using a stop-signal paradigm embedded within a pretest—activity—posttest protocol. As previously described, a stop-signal paradigm consists of a categorization task involving two sets of stimuli. In Experiment 1, the pupils’ task was to indicate whether an arrow was pointing to the right or to the left (i.e., Go trials). In a quarter of the trials, called the Stop trials, the appearance of a red square after the stimulus indicated to the participants that they must stop their motor response. The participant’s ability or inability to stop the motor response execution process is then used to calculate the time required for the inhibition process to operate and thus to assess their inhibitory abilities.

However, it is impossible to directly measure the time required for the inhibition process to operate for two reasons. First, unlike the motor response execution process, the behavioral inhibition process leaves no trace (Herrera Gomez, 2015). Second, there is a “point of no return” after which the motor response execution process becomes ballistic, that is irrepressible. Beyond this point, stopping the completion of the motor response is thought to be impossible (De Jong et al., 1990; Jennings, 1992). Thus, the inhibition of a preponderant response results from the intervention of both the inhibition process and the motor response execution process and more especially from the way in which these operate in relation to one another (Logan and Cowan, 1984; Logan, 1994; Verbruggen and Logan, 2008). In other words, an individual’s ability to inhibit their motor response depends on the delay between the stimulus that triggers the execution process (i.e., the arrow in our study) and the stimulus that triggers the inhibition process (i.e., the red square in our study). The longer the delay, the less likely the individual is to stop the execution process. This also means that the less time that is needed for the inhibition process to operate, the more this delay can be increased. The experimental principle of the stop-signal paradigm therefore consists in varying the duration of the delay (commonly called the Stop Signal Delay, or SSD) between the appearance of the stimulus triggering the motor response and the appearance of the stop signal in order to evaluate the time required for the inhibition process to operate. These variations in SSD and the participant’s ability or inability to stop the execution process can then be used to indirectly calculate the latency of the inhibition process. This latency is called the Stop Signal Response Time (SSRT) (Band et al., 2003) and correspond to the measure used to evaluation the efficiency of one’s inhibition process in a stop-signal paradigm. A low SSRT is an indication of efficient inhibition, and conversely a high SSRT is interpreted as a sign of inefficient inhibition.

Experiment 1 therefore aimed to assess the inhibition abilities of school-age participants through a calculation of their SSRTs and in particular to determine whether there was a variation in the latency of the inhibition process in participants after a knitting bout. Because SSRTs are considered stable in individuals (Band et al., 2003), the appearance of any intra-individual variability could be attributed to a behavioral adjustment to the task (Herrera Gomez, 2015) and/or to variations in the attentional abilities allocated to doing the task (Pessoa, 2009). A greater decrease in SSRT between the two times of testing was therefore expected for the pupils in the knitting group compared with those in the recess group.

### Methodology

#### Sample

Sixty-six children in 4th and 5th grades took part in this experiment. They were recruited from two elementary schools that came under the category of *Réseau d’Éducation Prioritaire* (priority education network^1^) in Paris’s 19th arrondissement. Informed consent was obtained from both parents and children after a presentation of the confidentiality and anonymity rules concerning the data to be collected. In order to ensure that the pupils did not present any cognitive, attentional or mnesic deficits, the WISC-V “Matrix Reasoning”, “Coding” and “Picture Span” subtests (Wechsler, 2014) were administered. The threshold value for inclusion in each of these tests was the lower limit value of the low mean of participants aged 11 years 3 months (maximum age of the sample). With reference to the values proposed by WISC-V, the threshold value was set at 10 for the “Matrix Reasoning” subtest, 27 for the “Coding” subtest and 16 for the “Picture Span” subtest. Eight pupils were excluded on the basis of the cognitive, attentional and mnesic abilities tests, and six pupils were excluded because they did not follow the instructions correctly when performing the stop-signal task (two did not complete the activity, and four were just pressing automatically). The final sample consisted of 52 pupils (20 boys and 32 girls). A power sensitivity analysis indicates that with this sample size, we would have been able to detect a minimal effect size (*η*^2^) of 0.042, given *α* = 0.05 and power (1–*β*) = 0.80. The pupils were randomly assigned to the two experimental groups, which differed according to the activity delivered between pretest and posttest (i.e., Knitting vs. Recess). A Student’s *t*-test was carried out to verify that the groups remained equivalent for each of the control tests (*t* < 1). The participants’ characteristics and their distribution in the experimental conditions are presented in Tables 1, 2.

**Table 1:** Characteristics of Experiment 1 sample as a function of grade and activity group.

Grade	Mean age (SD)	Age range	Gender	Knitting	Recess	*n*
4th	9 y. 6 m. (3 m.)	9 y. 0 m.–9 y. 11 m.	11 B /15 G	13	13	26
5th	10 y. 6 m. (3 m.)	10 y. 0 m.–10 y. 11 m.	9 B / 17 G	13	13	26
Total			20 B /32 G	26	26	52

**Table 2:** Control tests results as a function of grade and activity group in Experiment 1.

Tests		Mean score (SD)		Range	
		4th G	5th G	4th G	5th G
PIC	rec	23 (2)	22 (3)	18–26	16–26
	knit	22 (3)	25 (1)	16–26	22–26
MAT	rec	12 (2)	13 (2)	10–16	10–16
	knit	15 (2)	15 (1)	10–17	12–16
COD	rec	41 (4)	54 (9)	32–45	27–56
	knit	44 (9)	56 (13)	27–56	33–71

#### Materials and procedure

The experimental procedure was divided into three phases corresponding to the pretest-Activity-posttest design. In the first phase, the participants were invited to perform the stop-signal task. In the second phase, depending on the experimental group to which they had been assigned, the pupils either had recess in the school playground or they took part in a stand-alone knitting activity in a group of approximately five pupils. Both sessions lasted 20 min. In the knitting activity, the participants were asked to carry on with their knitting individually but were told they could communicate and help one another. In the third and final phase, following the knitting and recess sessions, the participants were asked to perform again a stop-signal task.

The stop-signal tasks took placed in a quiet room accompanied by the psychologist carrying out the experiment. Each participant was positioned in front of a computer screen situated approximately 50 cm away. The task had been constructed using Opensesame software (Mathôt et al., 2012) and was presented on DELL laptops with a 15.4″ screen size.

**Figure 1:**
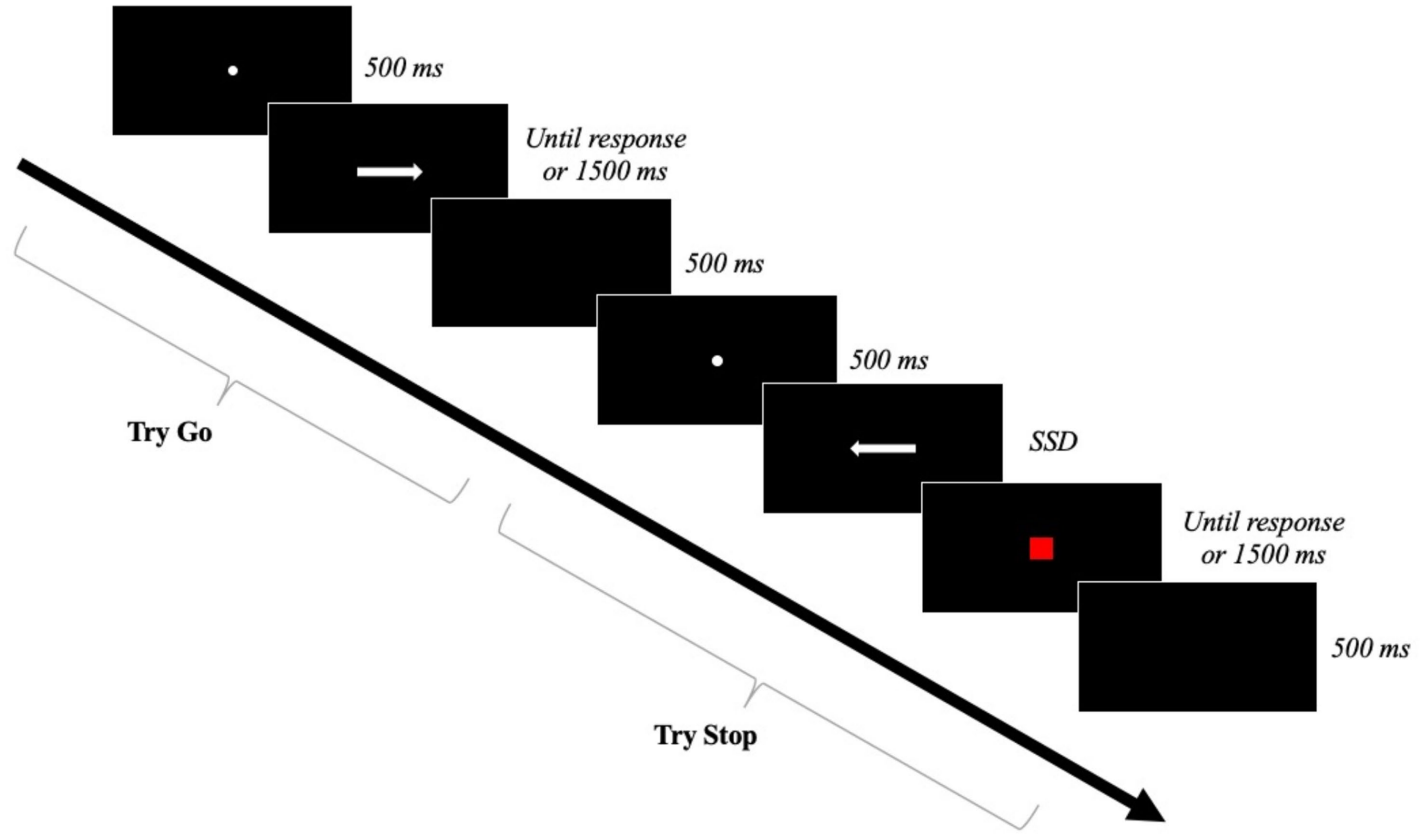
Stop-signal task of Experiment 1.

The pupils completed a categorization task in which the categorization criterion was the direction of an arrow presented on the screen. These arrows were 3 cm long and 1 cm wide and appeared pointing either right or left. The participants indicated their response by pressing the left arrow on the computer keyboard when the arrow pointed left and the right arrow when the arrow pointed right. Each participant was given an initial training block of 12 trials and then a total of 240 trials divided into 4 blocks of 60 trials. In a quarter of the experimental block trials, a 2 cm^2^ red square replaced the arrow, indicating to the participant that they had to stop their response (i.e., Stop trials). Figure 1 presents the procedure of the stop-signal task. Thus, each experimental block consisted of 48 Go trials and 12 Stop trials, randomized by the software. The training block consisted of 10 Go trials and 2 Stop trials. The participants were instructed to be as fast and accurate as possible and to stop their response when they saw the stop signal, represented by the red square. In addition, they were instructed not to wait for the potential appearance of the stop signal.

Each trial began with a fixation point in the center of the screen lasting 500 ms followed by the presentation of an arrow pointing either right or left. In the Go trials, the stimulus remained on the screen until the participant responded or, in the case of no response, for a maximum duration of 1,500 ms. There was a 500 ms inter-stimulus interval between each trial. In the Stop trials, a red square was displayed after the go signal (i.e., the arrow), with a SSD that varied from one trial to the next. The SSD varied according to a dynamic tracing procedure that adapted to the participant’s performance (Logan, 1994). Based on previous studies on a population of children of a similar age (Urben et al., 2014), the SSD was initially set at 250 ms. It was then increased or decreased by 50 ms at each *n* + 1 Stop trial depending on the participant’s success or failure in stopping their motor response in the *n* Stop trial. This procedure increases or decreases the difficulty of stopping the motor response at the next Stop trial. According to Band et al.’s (2003) mathematical model, the objective is to achieve a success rate close to 50% in the Stop trials for an optimal evaluation of the SSRT.

### Results

ANOVAs were conducted on the latency of the inhibition process as measured by the SSRTs. First, an ANOVA with 2 Activity levels (Knitting vs. Recess) × 2 Time of Testing levels (Pretest vs. Posttest) × 2 Grade levels (4th vs. 5th) was carried out. This first analysis indicated that there was no significant effect of Grade and that it did not interact with the other variables (always *F* < 1.85). Since we did not have a developmental hypothesis, this variable was combined in a second analysis, whose results are presented below. Table 3 gives the means and standard deviations of the different measures in the stop-signal task in Experiment 1.

**Table 3:** Performances in the stop-signal task as a function of time of testing and activity group in Experiment 1.

Measure	Knitting condition						Recess condition					
	Pretest		Posttest		Difference		Pretest		Posttest		Difference	
	Mean	SD	Mean	SD	Mean	SD	Mean	SD	Mean	SD	Mean	SD
GoRT	632.04	164.29	680.11	180.6	48.07	95.51	636.96	105.78	681.44	125.89	44.48	104.2
ACCgo	95.12	3.96	96.77	2.63	1.65	3.57	94.18	4.5	94.02	4.94	−0.16	3.34
ACCstop	58.73	12.26	65.06	12.78	6.33	7.56	60	9.01	62.87	9.87	2.87	8.26
SSRT	255.36	46.58	227.14	43.78	−28.22	51.39	239.64	55.53	243.23	56	3.59	52.56

GoRT, Go trials response times in ms; ACCgo, % of successful Go trials; ACCstop, % of successful stop trials; SSRT, stop signal reaction time in ms.

The latency of the inhibition process (i.e., SSRT) was calculated using Logan and Cowan’s (1984) procedure. Prior to starting this procedure, response times of less than 200 ms, considered to be reflex responses, and times greater than 2.5 standard deviations from the participant’s mean were removed (Urben et al., 2012). The removed data accounted for less than 3% of the total trial data.

Prior to conducting the ANOVA on the SSRTs thus obtained, Bravais-Pearson correlation analyses were carried out between the GoRT and SSRTs for each of the experimental groups at both times of testing in order to test for independence between the motor response execution process and the inhibition process. None of the analyses revealed a significant correlation between the two variables (always *r* < 0.289).

**Figure 2:**
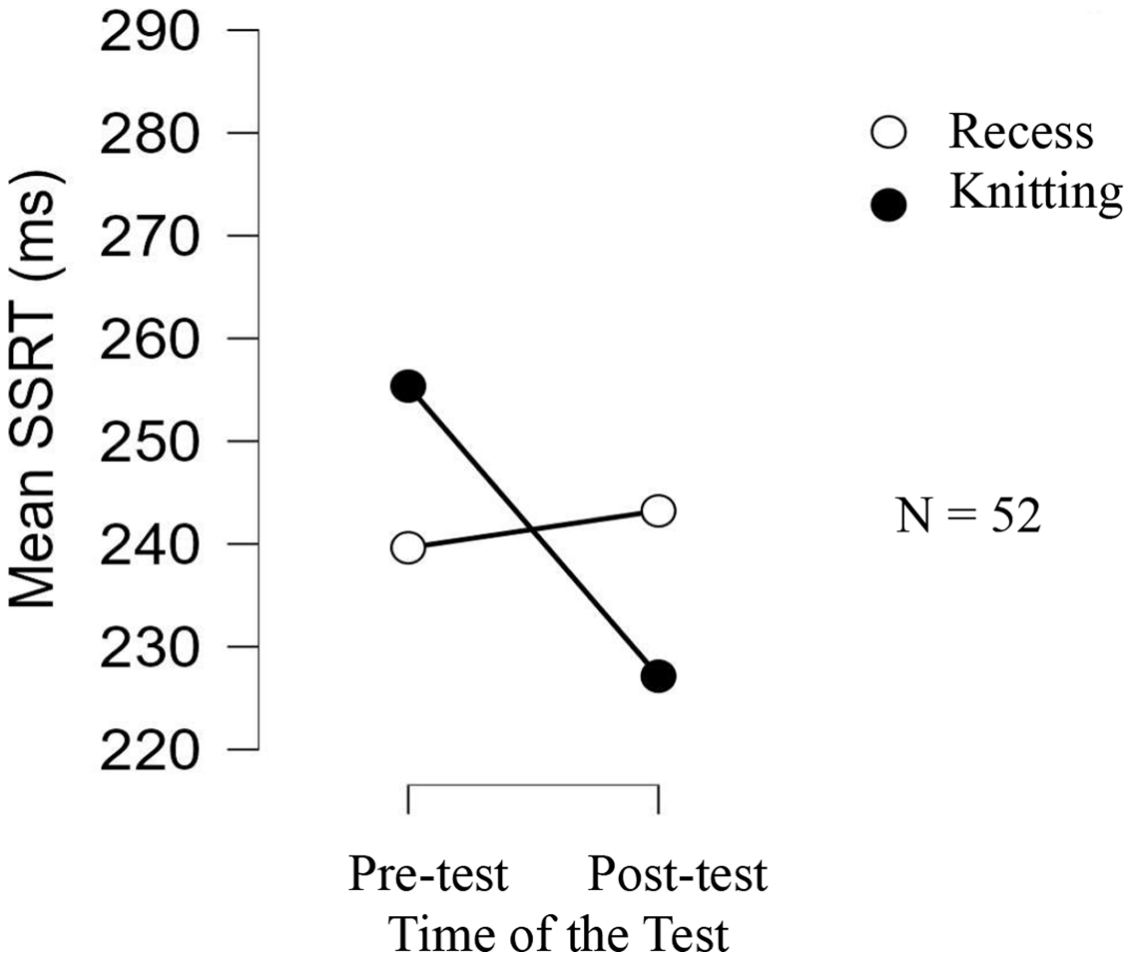
Mean of the SSRT of the two experimental groups in the cool stop-signal task as a function of the time of the test in Experiment 1.

The ANOVA conducted on the SSRTs showed neither an Activity nor a Time of Testing effect, but it did reveal an interaction between these two variables [*F*(1, 50) = 4.869, *p* = 0.032, *n*^2^ = 0.025]. The post-hoc tests showed a decrease in the SSRTs between pretest and posttest for the knitting group (*t* = 2.769, *p* = 0.038, *d* = 0.384), while no difference was observed for the recess group (*t* < 1). Those results are illustrated in Figure 2. No other differences reached the significance level.

An additional analysis was conducted on the Go trials response times (GoRT) to verify that the processing speeds in performing the categorization task were similar in the two experimental conditions. This ANOVA showed a Time of Testing effect [*F*(1, 50) = 11.145, *p* = 0.002, *n*^2^ = 0.025] but no significant effect of Activity or any interaction between these two variables.

Experiment 1 aimed to directly investigate the effects of a knitting session on the inhibition abilities of schoolchildren aged 9–11 using a cool stop-signal task. The results showed that the motor response and motor inhibition execution processes were independent and also, more importantly for our purposes, a decrease in latency for motor inhibition (i.e., SSRT) in the knitting group and no change in the recess group. Thus, the data indicate that the cool inhibition abilities of the pupils who took part to the knitting bout were optimized. Identical evolutions in the participants’ motor response execution process times (i.e., GoRT) between the two times of testing show a similar adaptation to the task by both experimental groups.

## Experiment 2

Experiment 2 was designed to explore further the beneficial effect of knitting on inhibitory abilities observed in Experiment 1. It aimed at examining whether the effect expanded into hot inhibitory capacities as well. Experiment 2 was based on Urben et al.’s (2012) study in which the emotional content of the stimuli was manipulated as the children undertook the stop-signal task. Their findings revealed that the emotional content introduced altered the participants’ inhibition abilities regardless of valence (i.e., positive or negative)—that is SSRTs were increased when the stimuli were emotionally charged compared to neutral ones. The rational of Experiment 2 was then that if the beneficial influence of knitting spread out to hot inhibitory abilities, it may reduce or eliminate the negative impact of emotionally charged stimuli on pupils’ inhibitory abilities. In Experiment 2, each participant performed a stop-signal task twice, once in each of two experimental conditions that were taken from Urben et al. (2012): a cool stop-signal task in which the participants had to categorize faces, which were all neutral in expression, according to sex (Female vs. Male) and a hot stop-signal task in which again the participants had to categorize faces, but this time the faces expressed an emotion of either sadness or joy, and the participants were asked to categorize the faces based on the emotion they expressed (sadness vs. joy). It is important to note that in the seminal work of Urben et al. (2012), there was an additional classical neutral condition similar to the one we used in Experiment 1 and that the results showed no difference in children inhibition abilities with the neutral emotional content. Prior to completion of both the stop-signal tasks, participants took part either in a knitting or a recess session. In the recess group, we expected identical results than those reported in Urben et al. study, that is, an increase in SSRTs for pupils when the faces expressed an emotion of sadness or joy compared with the neutral faces. As for the knitting group, we assumed that the results would depend on whether the beneficial impact of knitting expanded into hot inhibitory abilities or not. If it did, a reduced increase or no change for pupils in the knitting group was expected. This pattern of results would reflect in the appearance of a significant interaction between Activity (Knitting vs. Recess) and Emotional Charge of the Material (Neutral vs. Emotional). On the reversed, if the impact was restrained to cool inhibition abilities only, the knitting group would show a pattern of SSRTs identical to the recess group.

### Methodology

#### Sample

Forty-three children from grades 4th and 5th took part in this experiment. Their elementary school was located in Paris’s 19th arrondissement and categorized as *Réseau d’Éducation Prioritaire*. None of these pupils had participated in Experiments 1. The final sample consisted of 37 pupils (15 boys and 22 girls). Four pupils were excluded on the basis of tests measuring cognitive, attentional and mnesic abilities according to the same criteria as those applied in Experiments 1. Two additional pupils were not retained because they did not follow the instructions when carrying out the stop-signal task (they were not taking it seriously and just responding automatically). A power sensitivity analysis indicates that with this sample size, we would have been able to detect a minimal effect size (*η*^2^) of 0.054, given *α* = 0.05 and power (1–*β*) = 0.80. The participants were randomly assigned to the two experimental groups, which differed according to the activity that would be delivered before the test (i.e., Knitting vs. Recess). In order to ensure the comparability of the two groups, a Student’s *t*-test was conducted on the scores of the control subtests described in Experiment 1. The results showed no significant difference between the two groups for any of the subtests (always *t* < 1.377). Tables 4, 5 give the distribution of pupils as a function of the experimental conditions and group characteristics.

**Table 4:** Characteristics of Experiment 2 sample as a function of grade and activity group.

Grade	Mean age (SD)	Age range	Gender	Knitting	Recess	*n*
4th	9 y. 8 m. (4 m.)	9 y. 3 m.–10 y. 1 m.	8 B /11 G	9	10	19
5th	10 y. 7 m. (4 m.)	10 y. 0 m.–11 y. 2 m.	7 B / 11 G	10	8	18
Total			15 B /22 G	19	18	37

**Table 5:** Control tests results as a function of grade and activity group in Experiment 2.

Tests		Mean score (SD)		Range	
		4th G	5th G	4th G	5th G
PIC	rec	22 (4)	24 (2)	16–26	20–26
	knit	22 (3)	25 (1)	16–26	22–26
MAT	rec	13 (1)	13 (1)	11–16	11–15
	knit	13 (2)	13 (2)	10–15	10–16
COD	rec	38 (9)	44 (9)	27–53	30–57
	knit	39 (6)	52 (11)	30–46	34–53

#### Materials and procedure

The experimental procedure differed from that of Experiment 1 in that it was not a pretest-posttest design. The participants were asked to perform two stop-signal tasks, one on emotionally neutral content (i.e., cool stop-signal), and one on emotionally charged stimuli (i.e., hot stop-signal). Each participant was tested during two separate sessions, which were delivered a week apart in a quiet room accompanied by the psychologist conducting the experiment. Prior to completing each task, the participants had either a 20-min knitting bout or a recess session lasting a similar length of time. Each participant was positioned in front of a computer screen situated approximately 50 cm away. The task had been constructed using Opensesame software (Mathôt et al., 2012) and was presented on DELL laptops with a 15.4″ screen size. The stop-signal tasks followed the same procedure as in Experiment 1. Only two elements differed, namely the nature of the stimuli used and the criterion given to perform the categorization task. We therefore describe below only the information relating to these two elements. In the cool stop-signal task, the arrows in the Experiment 1 task were replaced by 12 cm × 9 cm rectangular images representing the faces of Caucasian men and women expressing an emotion described as neutral. A total of 30 faces were used, with an equal number of male and female faces. The participants completed a categorization task in which the categorization criterion was sex. They were instructed to indicate whether the face appearing on the screen was male or female. One half of the participants responded by pressing the A key, which had the letter H [for “homme” (male)] stuck on top of it, when a male face appeared and the P key, which had the letter F [for “femme” (female)] stuck on top, when a female face appeared. The keys were reversed for the other half of the participants.

**Figure 3:**
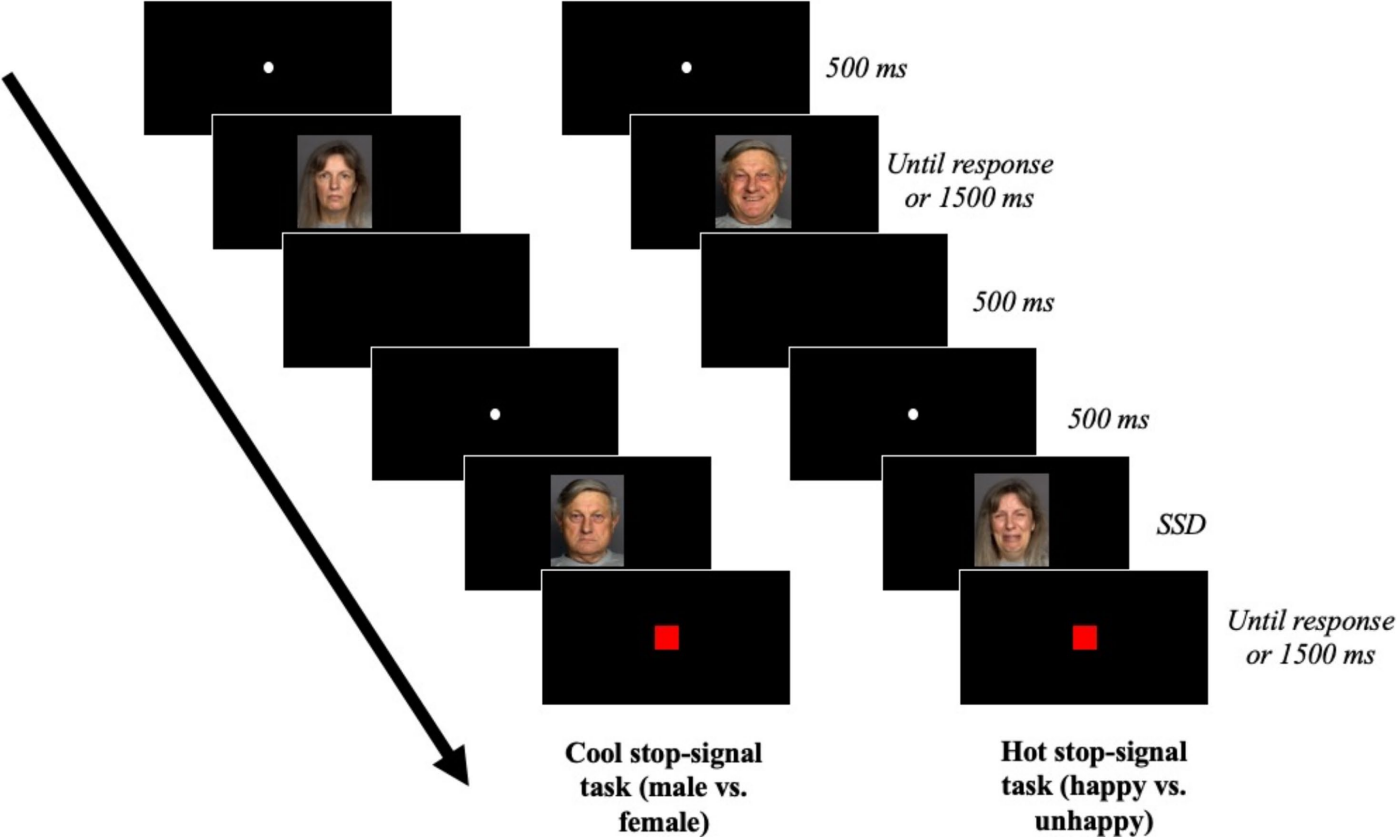
Stop-signal tasks of Experiment 2. Faces reproduced with permission from Max Planck Institute for Human Development, Center for Lifespan Psychology, Berlin, Germany, available from Ebner et al. (2018).

For the hot stop-signal task, the stimuli were images of the same dimensions representing faces expressing an emotion of sadness or joy. The faces were the same as those in the cool stop-signal task, hence this task comprised 60 different faces. The number of happy and sad faces was equal for both sexes. For this task, the categorization criterion was the emotion expressed by the face, so participants had to indicate whether the face presented was happy or sad. One half of the participants indicated their response by pressing the A key, which had a happy emoticon stuck on top of it, when a happy person’s face appeared, and on the P key, which had a sad emoticon stuck on top of it, when a sad person’s face appeared. The keys were reversed for the other half of the participants. The procedure of this hot-stop-signal task is illustrated in Figure 3.

All the faces used were drawn from the Max Planck Digital Library’s FACES experimental material (Ebner et al., 2018). The order in which the two tasks were administered (i.e., cool vs. hot) was counterbalanced within each of the experimental groups.

### Results

ANOVAs were conducted on the latency of the inhibition process as measured by the SSRTs. First, an ANOVA with 2 Activity levels (Knitting vs. Recess) × 2 Emotional Charge of the Material levels (Neutral vs. Emotional) × 2 Grade levels (4th vs. 5th) was carried out. This first analysis indicated no significant effect of the Grade variable and also that it did not interact with the other variables (always *F* < 1). Because we did not have a developmental hypothesis, this variable was therefore combined in a second analysis, whose results are presented below. Results of Experiment 2 are presented in Table 6.

**Table 6:** Performances in stop-signal task as a function of stimuli content and activity group in Experiment 2.

Measure	Neutral				Emotional				Difference			
	Knitting		Recess		Knitting		Recess		Knitting		Recess	
	Mean	SD	Mean	SD	Mean	SD	Mean	SD	Mean	SD	Mean	SD
GoRT	828.13	126.14	823.96	122.53	838.09	105.43	885.83	94.79	9.97	104.65	61.86	141.28
ACCgo	91.93	5.69	90.29	5.02	86.42	5.88	85.96	6.98	−5.51	7.02	−4.33	4.85
ACCstop	66.42	7.88	63.43	10.70	65.80	7.68	67.23	8.41	−0.62	8.09	3.80	7.44
SSRT	228.80	47.96	233.47	27.06	235.61	37.08	297.03	59.35	6.81	40.90	63.56	44.94

GoRT, Go trials response times in ms; ACCgo, % of successful Go trials; ACCstop, % of successful stop trials; SSRT, stop signal reaction time in ms.

The latency of the inhibition process (i.e., SSRT) was calculated using the same procedure as that applied in Experiment 1. The removed data accounted for less than 3% of the total trial data.

Prior to conducting the ANOVA, Bravais-Pearson correlation analyses were carried out between the GoRT and SSRTs for each of the experimental groups (Knitting vs. Recess) for each of the tasks (cool stop-signal vs. hot stop-signal) in order to test for independence between the motor response execution process and the inhibition process. None of the analyses revealed a significant correlation between the two variables (always *r* < 0.423).

**Figure 4:**
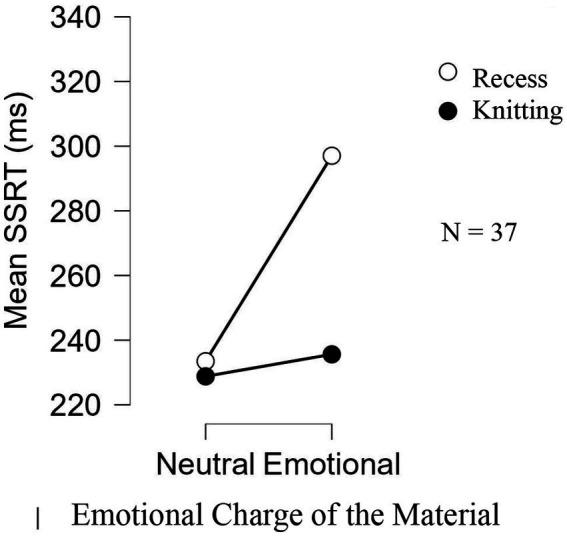
Mean of the SSRT of the two experimental groups as a function of the emotional intensity of the material in Experiment 2.

An ANOVA with 2 Activity levels (Knitting vs. Recess) × 2 Emotional Charge of the Material levels (Neutral vs. Emotional) × 2 Order of Administration levels (Neutral-Emotional vs. Emotional-Neutral) was conducted. The results showed an Activity effect [*F*(1, 33) = 5.655, *p* = 0.023, *n*^2^ = 0.137] and an Emotional Charge of the Material effect [*F*(1, 35) = 21.265, *p* < 0.001, *n*^2^ = 0.095]. In addition, as anticipated, they showed that these two variables interacted [*F*(1, 35) = 12.946, *p* = 0.001, *n*^2^ = 0.059]. The post-hoc tests confirmed an increase in SSRTs between the cool stop-signal task and the hot stop-signal task for the Recess group (*t* = 5.456, *p* < 0.001, d = 0.097), while no difference was observed for the Knitting group (*t* < 1). Similarly, the difference in SSRTs between the Knitting and Recess groups for the hot stop-signal task was significant (*t* = 3.785, *p* = 0.002, *d* = 0.622). Those results are illustrated in Figure 4. No other variable was found to be significant either on their own or in interaction.

The ANOVA conducted on the GoRT (i.e., Go trials response times) showed no significant variables either on their own or in interaction (*F*’s < 3.146).

The purpose of Experiment 2 was to examine whether the beneficial impact of knitting on cool inhibitory abilities observed in Experiment 1 expanded into hot inhibitory abilities as well. To achieve this objective, two stop-signal tasks were administered, one with neutral content, the other with emotional content. An effect of emotional content was observed in the pupils who had a recess session prior to completing the tasks as their SSRTs in the task with the emotional material were significantly higher than in the task with neutral content. However, this effect was found to disappear in the pupils who had taken part in the knitting session, their ability to inhibit a dominant response remaining stable between the two tasks. These results then demonstrate that an acute knitting boot optimizes pupils’ inhibition abilities even when processes operate on emotionally charged information.

## General discussion

The purpose of this research was to examine the effects of knitting on the cognitive sphere of school-age children. Considering together the likeness of knitting practice with AF meditation (Turney, 2009; Riley et al., 2013; Corkhill et al., 2014; Alhers and Thomas, 2019) and the influence of AF meditation on EF (Moore and Malinowski, 2009; Thurman and Torsney, 2014; Gallant, 2016; Kiani et al., 2017; Luu and Hall, 2017; Cásedas et al., 2019), two experiments were designed to test the assumption that knitting practice could optimized executive functions such as inhibition. Experiment 1 assessed the impact of an acute knitting bout on the pupils’ motor cool inhibition abilities using a cool stop-signal task, while Experiment 2 examined its influence using both a cool and a hot stop-signal tasks.

The results of Experiment 1 indicated a decrease in the latency of motor inhibition (i.e., SSRT) between the pretest and posttest measures among the pupils who had immediately before performing the post-test task, participated in the knitting boot but not among those who went to recess. In Experiment 2, while the emotional content of information resulted in an increase in SSRT for the pupils who had participated in the recess session prior to performing the task—as previously noted in the literature (Urben et al., 2012), this effect disappeared for those who had participated in the knitting bout. In addition, in both experiments, independence between the motor response execution processes and the inhibition processes mobilized in a stop-signal task was observed (Logan, 1994; Richard Ridderinkhof et al., 1999; Urben et al., 2014), and more importantly, no differences in motor response execution times (i.e., GoRT) between the two experimental groups appeared. This latter finding allows to interpret any decrease in the latency of motor inhibition (i.e., SSRT) as an optimization of the motor inhibition abilities, and vice versa for any increases in latency. The combined findings of the two experiments therefore demonstrate a beneficial effect of a prior knitting session on the pupils’ inhibition abilities whether cool or hot. While Experiment 1 showed an optimization of the pupils’ cool inhibition abilities immediately following the knitting session, Experiment 2 revealed that this positive impact of a knitting bout expands to hot inhibition processes as well. They thus raised the question as to why EF would be sensitive to knitting practice?

Three proposals can be issued for an answer to this question. The first two lie in the likeliness between knitting and attentional focused meditation claimed by some authors (Turney, 2009; Riley et al., 2013; Alhers and Thomas, 2019). First, Carter et al. (2005) showed that sustained attention abilities assessed by a binocular rivalry task were improved just after a 20-min session due to maintaining of the individuals’ attentional state. They argued that the focus on an object for a certain period of time had allowed the subjects to benefit from the optimization of their attentional capacities during the subsequent task. In another study, Wenk-Sormaz (2005) also reported an increase in performance of selective attention to a Stroop task following a FA meditation session. If the analogy between FA meditation and knitting in children is relevant, the beneficial effects of knitting on executive control observed in the presented studies could be the result of a maintenance of the “attentional state” of the pupils following the knitting activity. The second proposal would relate to an increase of attentional capacities of individuals not direct but mediated by a modulation of their emotional state. Indeed, another argument that has led some authors to compare meditation and knitting rests on the fact that both activities are thought to generate a similar state of stable emotional calm. The subjects testified that the practice of these activities give them positive emotions and allows them to distance themselves from negative emotions and the stress of everyday life (Ferber, 2005; Katz-Freiman, 2010; Riley et al., 2013; Alhers and Thomas, 2019). Consistent with those declarative data, Makowski et al. (2019) showed a faster disengagement of attention from emotional stimuli in their expert meditators than in their control group participants, leading to a rapid recovery of their attentional abilities. Similarly, the disappearance of the effect of emotional content on inhibition abilities observed in Experiment 2 could be explained by a faster processing of this emotional information and thus a faster reinvestment of attentional abilities for the task in hand. This idea would fit with models that explain how and why emotions impact cognitive processes such as Ellis and Moore’s (1999) Attentional Resource Allocation model, which postulates that emotions monopolize a proportion of the resources allocated to the task in hand, or the claim made by Pessoa (2009) that emotional intensity rather than valence generates interference with the processing in progress. If the level of intensity decreases, the resources allocated to processing these emotions are thought to then be freed up for diversion to the task. Thus, our results may suggest that the optimization of inhibition abilities following an acute knitting bout could be indirect and mediated by a modulation of the pupil’s emotional state.

A third proposal lies on the comparison of knitting not with meditation but with physical activity practice. There is a large body of evidence supporting the positive effects of physical activities—acute and chronic—on children’s EF and more specifically aerobic exercises (Best, 2010; Ludyga et al., 2016; Ishihara et al., 2021). Multiple—but not exclusive—explanations have been proposed to account for these effects, that may apply to knitting practice as well. First, it is assumed that the goal-directed problem-solving feature of physical activities may allow to develop skills similar to those require to perform EF tasks. The skills gained during physical activities would then transfer to EF tasks. The second pathway through which physical activities may facilitate EF is the execution of complex and fine motor movements as execution of these movements recruits neural circuitry associated with EF, that is the prefrontal neural circuitry. Finally, there is converging findings towards a priming effect of physical exercise as it promotes “chemical changes leading to an increased state of arousal that may enhance cognitive performance.” (Best, 2010, p. 342). Hence, it could be argued that in the present research, an acute knitting bout resulted in a similar phenomenon that may have increase attentional resources. At last, it should be noted that enhanced EF was also observed with other activities involving both attention and motor skills such as music (Colombo et al., 2020) or dance (Shen et al., 2020). All together, this may suggest that any activity that requires EF train them, and an acute phase of these activities may transfer into improved performance in tasks that require EF.

Before to conclude, a methodological issue inherent in the type of research we conducted merits discussion: the choice of the control group. In this matter, it seemed important to take into account the research’s environmental aspect and its anchoring in the children’s reality. It was thus apparent that recess was an appropriate activity for the control group because it is perceived as fun time out from classroom learning. It is used by teachers to provide a break in the sequence of cognitive activities engaged in during the school day (Delalande, 2009) and is intended to prevent a state of cognitive overload and promote subsequent learning. The results of the present study thus provide some information on both knitting practice and recess at school. They indicate that a knitting session is more effective at inducing a state that is conducive to cognitive activities than a recess session. There is no suggestion here, however, that group recess sessions outdoors should be replaced by knitting sessions, but the sequence of sessions in school timetables merits further investigation.

In conclusion, this research is the first to have examined the acute effects of knitting practice on schoolchildren’s EF. The results indicate an improvement in both cool and hot inhibition abilities, that is when inhibition operates either on neutral (i.e., Experiment 1) or emotionally charged stimuli (i.e., Experiment 2). More research is needed to examine whether an acute knitting boot may impact other executive functions, such as mental flexibility and the updating of working memory, as it has been observed for meditation (Moore and Malinowski, 2009; Thurman and Torsney, 2014; Gallant, 2016; Kiani et al., 2017; Luu and Hall, 2017; Cásedas et al., 2019). In addition, in keeping with the studies on aerobic exercise, determining whether EF is more sensitive to knitting practice when individuals undergo developmental changes needs to be clarified. Finally, the effects of chronic knitting practice in the school setting also merit investigation. From a more didactic perspective, the presented findings should encourage all those concerned with educational issues to re-consider the role and potential influence of manual activities at school.

## Data availability statement

The raw data supporting the conclusions of this article will be made available by the authors, without undue reservation.

## Ethics statement

Ethical review and approval was not required for the study on human participants in accordance with the local legislation and institutional requirements. Written informed consent to participate in this study was provided by the participants’ legal guardian/next of kin.

## Author contributions

FS prepared the data and wrote the manuscript. EL helped for data collecting and data analysing. SG wrote the manuscript and planed the whole work. All authors contributed to the article and approved the submitted version.

## Conflict of interest

The authors declare that the research was conducted in the absence of any commercial or financial relationships that could be construed as a potential conflict of interest.

## Publisher’s note

All claims expressed in this article are solely those of the authors and do not necessarily represent those of their affiliated organizations, or those of the publisher, the editors and the reviewers. Any product that may be evaluated in this article, or claim that may be made by its manufacturer, is not guaranteed or endorsed by the publisher.

## Footnotes

^1^The priority education policy aims to redress the impact of social and economic inequalities on academic success by reinforcing pedagogical and educational action in schools and institutions located in areas experiencing the greatest social difficulties (Ministère de l’Education Nationale, de la Jeunesse et des Sports, Circular No. 97-233 of 10/31/1997).
